# Determinants of Severe Nocturnal Hypoxemia in Adults with Chronic Thromboembolic Pulmonary Hypertension and Sleep-Related Breathing Disorders

**DOI:** 10.3390/jcm12144639

**Published:** 2023-07-12

**Authors:** Caner Çınar, Şehnaz Olgun Yıldızeli, Baran Balcan, Bedrettin Yıldızeli, Bülent Mutlu, Yüksel Peker

**Affiliations:** 1 Department of Pulmonary Medicine, School of Medicine, Marmara University, Istanbul 34854, Turkey; drcanercinar@gmail.com (C.Ç.); drsehnazolgun@yahoo.com (Ş.O.Y.); 2 Department of Pulmonary Medicine, School of Medicine, Koç University, Istanbul 34450, Turkey; drbaranbalcan@gmail.com; 3 Department of Thoracic Surgery, School of Medicine, Marmara University, Istanbul 34854, Turkey; byildizeli@marmara.edu.tr; 4 Department of Cardiology, School of Medicine, Marmara University, Istanbul 34854, Turkey; bulent.mutlu@gmail.com; 5 Department of Molecular and Clinical Medicine, University of Gothenburg, 405 30 Gothenburg, Sweden; 6 Department of Respiratory Medicine and Allergology, Faculty of Medicine, Lund University, 221 00 Lund, Sweden; 7 Division of Sleep and Circadian Disorders, Brigham and Women’s Hospital, Harvard Medical School, Boston, MA 02115, USA; 8 Division of Pulmonary, Allergy, and Critical Care Medicine, School of Medicine, University of Pittsburgh, Pittsburgh, PA 15260, USA

**Keywords:** CTEPH, pulmonary hypertension, pulmonary endarterectomy, sleep-related breathing disorders, nocturnal hypoxemia

## Abstract

Objectives: We aimed to investigate the occurrence of sleep-related breathing disorders (SRBDs) in patients with chronic thromboembolic pulmonary hypertension (CTEPH) and addressed the effect of pulmonary hemodynamics and SRBD indices on the severity of nocturnal hypoxemia (NH). Methods: An overnight polysomnography (PSG) was conducted in patients with CTEPH, who were eligible for pulmonary endarterectomy. Pulmonary hemodynamics (mean pulmonary arterial pressure (mPAP), pulmonary arterial wedge pressure (PAWP), pulmonary vascular resistance (PVR) measured with right heart catheterization (RHC)), PSG variables (apnea–hypopnea index (AHI)), lung function and carbon monoxide diffusion capacity (DLCO) values, as well as demographics and comorbidities were entered into a logistic regression model to address the determinants of severe NH (nocturnal oxyhemoglobin saturation (SpO_2_) < 90% under >20% of total sleep time (TST)). SRBDs were defined as obstructive sleep apnea (OSA; as an AHI ≥ 15 events/h), central sleep apnea with Cheyne–Stokes respiration (CSA–CSR; CSR pattern ≥ 50% of TST), obesity hypoventilation syndrome (OHS), and isolated sleep-related hypoxemia (ISRH; SpO_2_ < 88% under >5 min without OSA, CSA, or OHS). Results: In all, 50 consecutive patients (34 men and 16 women; mean age 54.0 (SD 15.1) years) were included. The average mPAP was 43.8 (SD 16.8) mmHg. SRBD was observed in 40 (80%) patients, of whom 27 had OSA, 2 CSA–CSR, and 11 ISRH. None had OHS. Severe NH was observed in 31 (62%) patients. Among the variables tested, age (odds ratio (OR) 1.08, 95% confidence interval [CI] 1.01–1.15; *p* = 0.031), mPAP (OR 1.11 [95% CI 1.02–1.12; *p* = 0.012]), and AHI (OR 1.17 [95% CI 1.02–1.35; *p* = 0.031]) were independent determinants of severe NH. Conclusions: Severe NH is highly prevalent in patients with CTEPH. Early screening for SRBDs and intervention with nocturnal supplemental oxygen and/or positive airway pressure as well as pulmonary endarterectomy may reduce adverse outcomes in patients with CTEPH.

## 1. Introduction

Pulmonary hypertension (PH) has three main types, pre-capillary PH, post-capillary PH, and combined pre-capillary and post-capillary PH, and it is based on mean pulmonary arterial pressure (PAP) > 20 mmHg measured with a right heart catheterization (RHC) [1]. Pulmonary arterial wedge pressure (PAWP) ≤ 15 mmHg and pulmonary vascular resistance (PVR) ≥ 3 Wood units are other acknowledged measures of the PH [1]. The current classification acknowledges five groups of PH, and Group 1 is pulmonary arterial hypertension (PAH), which is a rare condition with a prevalence of around 15 to 50 patients per million in Europe and the United States [1]. The average age for PAH diagnosis is rising, and older patients are increasingly being diagnosed with PAH. In the US-based Registry to Evaluate Early and Long-term PAH disease management (REVEAL), the mean age at diagnosis was 50 years, and five percent of the 2599 patients included were diagnosed at or after the age of 75 [2]. The French PH registry reported an average age of 50 years at diagnosis [3], whereas the “Comparative, Prospective Registry of Newly Initiated Therapies for Pulmonary Hypertension” (COMPERA) registry reported a mean age of 71 years [4]. Compared to younger patients, a higher percentage of patients experienced comorbidities as the patients’ ages advanced.

Chronic thromboembolic pulmonary hypertension (CTEPH) is mainly defined as a pre-capillary PH and classed as a Group IV PH. It was reported that 0.1–9.1% of individuals with pulmonary embolism develop CTEPH within two years after the initial diagnosis [5,6,7], and CTEPH is the only PH category that has a chance of being cured, mainly by pulmonary endarterectomy [8,9].

Sleep-related breathing disorders (SRBD) are defined as obstructive sleep apnea (OSA) disorders, central sleep apnea (CSA) syndromes, sleep-related hypoventilation disorders, and sleep-related hypoxemia [10]. An SRBD may also lead to an increase in PAP primarily during sleep and cause nocturnal hypoxemia [11]. Although SRBDs were reported in patients with pre-capillary PH, most of the studies included patients with idiopathic PAH [2,12,13]. Notably, OSA was one of the most prevalent comorbidities at the time of enrollment in the REVEAL registry, where 20% of the entire cohort had OSA [2]. Although the cause-and-effect relationship between pre-capillary PH and SRBDs is uncertain, it is known that mPAP may increase during sleep in patients with OSA [14].

Less is known regarding the occurrence of SRBDs in CTEPH. In a small study including 38 individuals with PH, 15 had CTEPH and 68% of the cohort had NH (oxygen saturation < 90% during at least 10%) [13]. In another study including 46 patients with stable idiopathic PAH (n = 29) and CTEPH (n = 17), NH was observed among 38 (82.6%) of them [15]. A later study found OSA, defined as an apnea–hypopnea index (AHI) ≥ 5 events/h, on a cardiorespiratory device in 32 out of 57 patients (56.1%) with CTEPH and suggested that a cardiac index was the most important parameter indicating the coexistence of OSA and CTEPH [16]. No information was available regarding the other SRBD subgroups and variables associated with the NH [16]. In another study, an unattended cardiorespiratory recording was conducted the night before and one month after elective pulmonary endarterectomy in 50 cases with CTEPH, and the occurrence of an SRBD (AHI ≥ 5 events/h) was reported among 32 (64%) patients, of whom 22 had OSA and 10 CSA, respectively [17]. One month after the surgical intervention, an SRBD was found among 34 (68%) patients, of whom 30 had OSA and 4 CSA. The authors concluded that CTEPH may trigger CSA but not OSA, and OSA may play a role in the development of CTEPH [17].

In the current study, we aimed to investigate the occurrence of SRBDs in a consecutive cohort of CTEPH, with patients who were on the waiting list for pulmonary endarterectomy. We also addressed to what extent pulmonary hemodynamics and SRBD indices determine the severity of NH in patients with CTEPH.

## 2. Material and Methods

### 2.1. Study Participants

**Figure 1:**
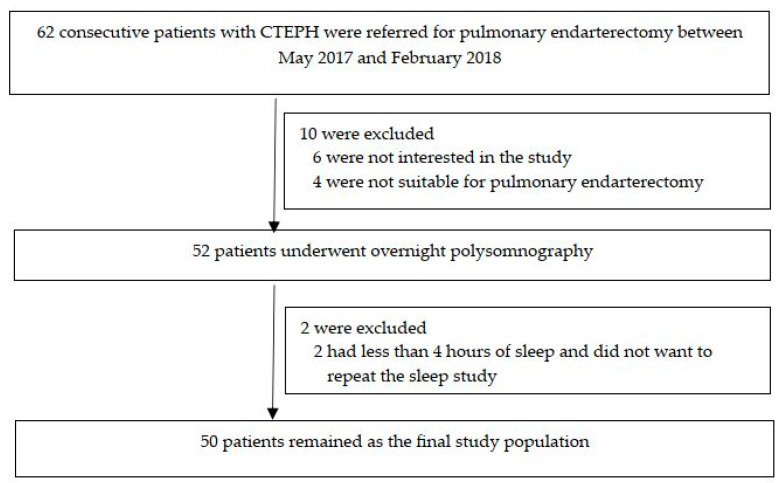
Flow chart of the study participants. Abbreviations: CTEPH, chronic thromboembolic pulmonary hypertension.

As illustrated in Figure 1, 62 cases older than 18 years were referred to the Pulmonary Hypertension Center of the Marmara University Pendik Education and Research Hospital between 5 May 2017 and 7 February 2018 for pulmonary endarterectomy. All participants underwent a computerized pulmonary angiography and RHC after that mismatch of perfusion defects detected in ventilation/perfusion scintigraphy (V/Q) despite 3 months of anticoagulant therapy. Patients with an mPAP > 20 mmHg measured with RHC were accepted as having CTEPH; PAWP ≤ 15 mmHg and PVR ≥ 3 Wood Units were additional measurements suggestive of CTEPH.

SRBDs were defined according to the International Classification of Sleep Disorders (ICSD)-10 as obstructive sleep apnea (OSA; as an AHI ≥ 15 events/h), central sleep apnea with Cheyne–Stokes respiration (CSA–CSR; CSR pattern ≥ 50% of total sleep time (TST)), obesity hypoventilation syndrome (OHS), and isolated sleep-related hypoxemia (ISRH)(nocturnal oxyhemoglobin saturation (SpO_2_) < 88% for 5 min or more without OSA, CSA, or OHS). Severe NH was defined as SpO_2_ < 90% for more than 20% of total sleep time (TST).

Two cases diagnosed with sarcoma and vasculitis, respectively, were considered as not suitable for the study, and two cases with a total sleep time of less than 4 h on PSG were excluded from the study.

### 2.2. Clinical Data Collection

Demographic data, body mass index (BMI), comorbid conditions, medications, supplementary oxygen treatment, preoperative echocardiography findings, RHC measurements, 6 min walk distance (SMWD), pulmonary function test, as well as carbon monoxide diffusion test (DLCO) measurements were recorded.

### 2.3. Pulmonary Function Testing

MIR Spirolab II spirometry (Medical International Research, Rome, Italy) was used to test pulmonary function, including forced expiratory volume in one second (FEV1) and forced vital capacity (FVC) [18], diffusing capacity of the lung for DLCO in a body plethysmograph (CareFusion Type MasterScreen PFT, Hoechberg, Germany), and performance of an SMWD. All results were assessed in accordance with ATS recommendations [18,19,20,21].

### 2.4. Transthoracic Echocardiography (TTE) and Right Heart Catheterization (RHC)

All study participants underwent detailed TTE based on the guidelines of the device (Epiq 7, Philips Healthcare, Andover, MA, USA) with a 3.5 MHz (S5-1) transducer. Digitally stored images were analyzed offline (Xcelera, Philips). Based on the guidelines, the systolic and diastolic characteristics of the left and right heart were measured as recommended [22,23]. Tricuspid regurgitation velocity and other echocardiographic signs were combined to assess the probability of PH as recommended in ERS guidelines [24]. RHC was performed following the overnight fast. The hemodynamic variables measured at end-expiration included mPAP and PAWP. Cardiac output was determined by the indirect Fick principle. PVR was calculated by dividing (mPAP−PAWP) by cardiac output (Wood Unit).

### 2.5. Polysomnography (PSG)

All patients were hospitalized for polysomnographic monitoring using the NOX-A1 system (Nox Medical Inc., Reykjavik, Iceland). The PSG recording included an electroencephalogram (F4/M1, F3/M2, C4/M1, C3/M2, O2/M1, O1/M2), electro-oculogram, submental and tibialis electromyograms, as well as an electrocardiogram. Ventilatory monitoring included a nasal pressure detector using a nasal cannula/pressure transducer system and thoracoabdominal movement detection through two respiratory inductance plethysmography belts. A finger pulse oximeter detecting heart rate, SpO_2_, as well as body position and movement detection were also included. Participants with a total sleep time of less than 4 h were offered a new PSG. Sleep stages and arousals were scored based on 30 s epochs in accordance with *The AASM Manual for the Scoring of Sleep and Associated Events* 2.5 [25] published by the American Academy of Sleep Medicine (AASM), independently of the patients’ clinics, by a certified sleep physician. Apnea was defined as an almost complete (≥90%) cessation of airflow, and hypopnea was defined as a decrease in nasal pressure amplitude of 30% or more and/or thoracoabdominal movement of at least 30% for at least 10 s if there was a significant oxyhemoglobin desaturation (reduction of at least 3% from the immediately preceding baseline value) and/or arousal, according to the latest recommendations of the AASM [25]. Furthermore, the total number of significant desaturations was scored, and the oxygen desaturation index (ODI) was calculated as the number of significant desaturations per hour of total sleep time. Minimum SpO_2_ and time spent below 90% SpO_2_ (TS90%) values were also recorded.

### 2.6. Statistics

All statistical analyses were performed using SPSS^®^ 26.0 for Windows^®^ (SPSS Inc., Chicago, IL, USA). The normality assumptions for all variables were made with the Shapiro–Wilk test. Continuous variables were reported as a mean with standard deviation or median with interquartile ranges (IQRs), and categorical variables were reported as percentages. Categorical variables were compared with Pearson’s Chi-Square Test, or when appropriate, Fisher’s Exact Test. The Mann–Whitney U Test was used when evaluating non-normally distributed (nonparametric) variables between two groups. The Spearman Correlation Test was used in the analysis of the measurement data with each other. Pulmonary hemodynamics (mPAP, PAWP, and PVR, measured with RHC as well as the echocardiographic sPAP, and PSG variables (AHI)), demographics, lung function tests, and DLCO values were entered into a logistic regression model to address the determinants of severe NH. Unadjusted and adjusted odd ratios (ORs) with 95% confidence intervals (CIs) for all variables associated with severe NH were performed, respectively. Age, sex, BMI, and significant variables associated with severe NH in univariate analysis were entered into the multivariate model. All statistical tests were two-sided, and a *p*-value < 0.05 was considered significant.

## 3. Results

As shown in Figure 1, 62 consecutive patients with CTEPH who were considered for pulmonary endarterectomy were eligible for the current study. Eight patients were not included, six of them were not interested in the study, and four were not suitable for endarterectomy. A total of 52 Patients underwent a one-night polysomnography. Two patients were excluded due to insufficient total sleep time and an unwillingness to repeat the sleep study. Thus, 50 patients (34 men and 16 women; mean age 54.0 (SD 15.1) years; mPAP 43.8 (SD 16.8) mmHg, mean daytime SpO_2_ 92.5 (SD 4.6) %; 20 (40.0%) on pulmonary vasodilators) were included (Figure 1).

**Figure 2:**
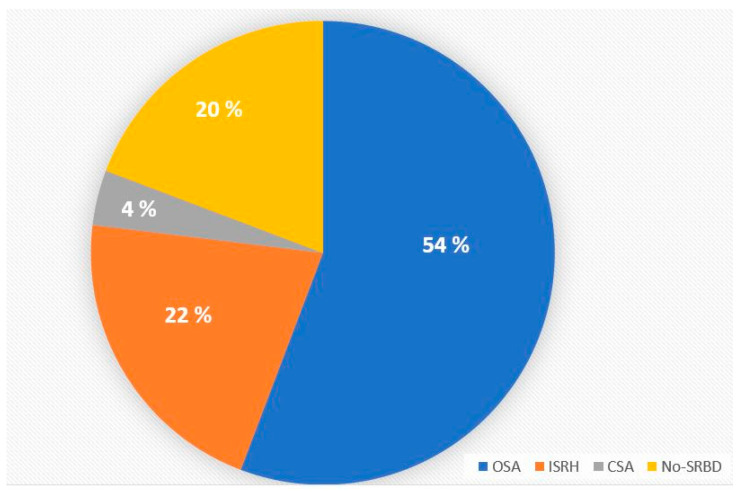
Distribution of SRBDs among patients with CTEPH. Definition of abbreviations: CSA, central sleep apnea; CTEPH, chronic thromboembolic pulmonary hypertension; ISRH, isolated sleep-related hypoxemia; SRBD, sleep-related breathing disorders.

As illustrated in Figure 2, SRBDs were observed among 40 (80.0%) patients in the entire cohort. None of the participants had OHS. Severe NH was observed in 31 (62%) of them.

As shown in Table 1, patients with severe NH were significantly older than the patients without severe NH. Sex distribution, body mass index (BMI), smoking history, as well as concomitant diabetes, systemic hypertension, and cardiac diseases were similar in both groups whereas asthma or chronic obstructive pulmonary disease was more common in the severe NH group. The proportion of patients on pulmonary vasodilator agents as well as on long-term oxygen therapy (LTOT) was slightly more common among the patients with severe NH, but the differences were not statistically significant. The SMWD test, WHO function class, ESS scores, and FEV1/FVC values were similar in both groups while DLCO tended to be lower in the NH group (Table 1).

As shown in Table 2, the estimated values for mPAP and PVR measurement were significantly higher in the NH group. Other RHC measurements including PAWP, CO, and CI did not differ significantly (Table 2).

Out of the 31 patients with severe NH, 21 (67.7%) had OSA, 8 (25.8%) had ISRH, and 2 (6.5%) had CSA with Cheyne–Stokes Respiration (CSA–CSR) whereas 6 out of 19 (31.6%) had OSA, and 3 (15.8%) had ISRH among the patients with no NH (*p* < 0.001). None of the patients without NH demonstrated CSA–CSR and OHS. As shown in Table 2, AHI and oxygenation parameters were expectedly more severe among the NH group whereas the total sleep time and proportion of N3 and REM sleep stages did not differ significantly. As illustrated in Table 3, the patients with ISRH had worse pulmonary hemodynamics regarding the mPAP and PVR measurements compared to the values among the OSA patients.

As shown in Table 4, unadjusted variables associated with severe NH were age, concomitant asthma or COPD, OSA and OSA indices, AHI and ODI, mPAP, and PVR. No significant correlation was found between mPAP and AHI.

In the multivariate logistic regression analyses, mPAP and AHI were independent determinants of severe NH, adjusted for age, BMI, sex, and concomitant asthma or COPD (Table 5). PVR was also associated with severe nocturnal hypoxemia in the multivariate analysis adjusted for AHI, age, BMI, sex, and concomitant asthma or COPD (OR 1.32 [95% CI 1.05–1.62; *p* = 0.019]).

## 4. Discussion

The current study showed that 80% of the patients with CTEPH had an SRBD, of whom 67.7% had OSA and 25.8% ISRH. Severe NH was observed among 62% of the entire cohort. In the multivariate linear regression models, the severity of the PH in terms of PAP levels as well as the severity of OSA in terms of AHI, significantly and independently from each other, determined the severity of the NH.

To our best knowledge, this is the first study to describe the occurrence of the SRBD subgroups based on PSG in a consecutive cohort of patients with CTEPH verified with RHC. A few previous studies on patients with CTEPH had smaller sample sizes, constituted subgroups of heterogenous clinical cohorts of PH, did not address the occurrence of SRBD subgroups, and not all sleep recordings were based on full overnight PSG, which have all limited the generalizability of the reports. Moreover, the severity and the determinants of the NH, which is an important prognostic factor in this high-risk group, have not yet been addressed accurately in previous studies.

SRBDs were shown to be prevalent in patients with PH in previous small studies. Schulz et al. found periodic breathing in 6 out of 20 consecutive patients with PH who were admitted for the pharmacological investigation of pulmonary vasoreactivity [26]. The patients with periodic breathing had more severe hemodynamic disturbances and greater hypoxemia than those without [26]. Minai et al. included 43 patients with idiopathic PAH or PAH connected to connective tissue illness (CTD–PAH) in a pulse oximetry study and reported that 30 (70%) of these patients had oxygen saturation <90% for more than 10% of the night [27]. The pulmonary hemodynamics of the nocturnal desaturators were more severe, and they were older than the patients with less or no desaturators; only one patient had significant sleep apnea [27]. In a PSG-based sleep study, Prisco et al. [28] included 28 patients, of whom 32% had idiopathic PAH and 68% with PAH from other etiologies. The mean AHI was 11.4 ± 19.8/h in the entire cohort, and 14 patients (50%) had an AHI ≥ 5/h. In contrast to our findings in the CTEPH cohort, the authors reported a significant association of AHI and time spent below 90% oxygen saturation with mPAP values in a heterogenous PH cohort [28].

The first sleep study in a larger heterogenous PH cohort (n = 169) using cardiorespiratory polygraphy was conducted by Dumitrascu et al. [29]. The researchers applied an AHI cut-off 10/h value for SRBD diagnosis and found OSA among 27 (16.0%) and CSA among 18 (10.6%). Male sex and higher BMI were the main characteristics of OSA patients whereas CSA patients were older, more hypocapnic, and had worse pulmonary hemodynamics than the PH patients without CSA [29]. In another retrospective study, Minic et al. reported the occurrence of an SRBD among 71% of 52 PH patients, of whom 56% had OSA and 44% had CSA [30]. No differences in cardiopulmonary hemodynamics or survival between those with and without SRBDs were found, and moderate to severe NH was not addressed specifically in that cohort [30]. In a larger PH cohort (n = 151), the occurrence of SRBDs was assessed with a simplified polygraphy cut-off AHI value of 5 events/h [31]. OSA was found in 29 patients (19.2%) and CSA in 29 (19.2%), and 32 patients (21.2%) died during an average follow-up of 1170 ± 763 days. The authors found no significant difference between PH patients with vs. without SRBDs regarding the mortality rates whereas NH was the only independent variable related to death in a multivariate Cox proportional hazards analysis [31].

Very few data exist in the literature regarding the occurrence of SRBDs in patients with CTEPH. Ulrich et al. [13] included 15 cases with CTEPH among 38 patients with PH, of whom 68% had NH defined as an oxygen saturation < 90% during at least 10% [13]. PSG was conducted among 22 patients, and most of the patients reported to have CSA whereas OSA was observed among only four cases [13]. In another small study, Jilvan et al. included 17 patients with CTEPH among 46 PH patients, of whom 38 (82.6%) had NH [15]. A later study [16] found OSA, defined as an AHI ≥ 5 events/h on a cardiorespiratory device, in 32 out of 57 cases with CTEPH (56.1%) and suggested that the cardiac index was the most important parameter indicating the coexistence of OSA. No information was available regarding the other SRBD subgroups and variables associated with the NH [16]. In another study by Rovere et al. [17], 32 cases were reported to have SRBDs (AHI ≥ 5 events/h) on a cardiorespiratory polygraphy among patients with CTEPH (64%), of whom 22 had OSA and 10 CSA. One month after pulmonary endarterectomy, SRBDs were prevalent among 34 (68%) patients, of whom 30 had OSA and 4 CSA. In contrary to their findings, the occurrence of CSA was only among two cases in our cohort. There was no report regarding the occurrence of severe NH before and after the surgical intervention in that study [17].

Several possible mechanisms were proposed for the association between SRBDs and PH. Severe NH, hypercapnia, and a rise in intrathoracic pressure result in changes in vascular tone and cardiac output, which may lead to an increase in mPAP [11]. Due to sympathetic overactivation, increased oxidative stress, systemic inflammation, and endothelial dysfunction, other cardiovascular diseases (hypertension, coronary artery disease, heart failure, and arrhythmias) are also common among patients with SRBDs [32]. An increase in the frequency of venous thromboembolism and pulmonary embolism in these individuals was also reported [33,34].

It is unclear exactly what causes these different SRBDs to manifest in people with PH or CTEPH. Rapid eye movement (REM) sleep and stage N3 have lower minute ventilation, which could be a factor in the increased ventilation/perfusion mismatch [15,35]. OSA is a relatively common disorder in people in their fourth, fifth, and sixth decades of life [36], and it was suggested that the finding by chance in a group of patients with an average age of 50–55 years is not unexpected [2,3]. Moreover, patients’ predispositions to arouse while experiencing respiratory control instability and the (moderate) reduction in lung volumes [35] were also suggested as possible contributory factors [37,38]. In patients with right heart failure, the upper airway dilator muscles may also be affected [39]. Furthermore, fluid retention and fluid shift in patients with PH, especially in elderly subjects, is another potential mechanism that may be involved in the occurrence of OSA in these patients [40]. Reactive hyperventilation in response to hypoxemia and enhanced chemosensitivity [41] may cause hypocapnia, which is thought to cause central apneas by lowering PaCO_2_ levels below the apneic threshold [42]. OSA is the most common SRBD found in CTEPH patients [17,43].

## 5. Conclusions

Severe NH is highly prevalent in patients with CTEPH. Early sleep monitoring and intervention with nocturnal supplemental oxygen and/or positive airway pressure as well as pulmonary endarterectomy may reduce adverse outcomes of NH in patients with CTEPH.

## Figures and Tables

**Table 1:** Demographic and clinical characteristics of the study population (*n* = 50).

	TS90% < 20% *n* = 19	TS90% ≥ 20% *n* = 31	*p* Value
Age, years	46.2 ± 14.1	58.7 ± 14.0	0.003
Male sex, n (%)	13 (68.4)	23 (69.7)	0.960
BMI, kg/m^2^	28.3 ± 4.4	28.1 ± 5.1	0.841
Obesity, n (%)	5 (26.3)	10 (32.3)	0.656
Current smoker, n (%)	1 (1.9)	2 (4.0)	1.000
Asthma or COPD, n (%)	1 (5.3)	11 (35.5)	0.018
Diabetes mellitus, n (%)	2 (10.5)	7 (22.6)	0.452
Hypertension, n (%)	5 (26.3)	14 (45.2)	0.237
Coronary heart disease, n (%)	4 (21.1)	5 (16.1)	0.715
Valvular heart disease, n (%)	1 (5.3)	4 (12.9)	0.637
Atrial fibrillation, n (%)	3 (15.8)	4 (12.9)	1.000
Pulmonary vasodilator use, n (%)	8 (15.4)	12 (38.7)	0.812
Awake SpO_2_, %	93.9 ± 2.0	88.9 ± 4.7	<0.001
LTOT, n (%)	1 (5.3)	5 (16.7)	0.384
SMWD, meter	327 ± 133	309 ± 101	0.219
WHO function class	1.89 ± 0.66	1.97 ± 0.75	0.729
ESS score	5.6 ± 3.9	5.2 ± 3.7	0.753
DLCO < 80%, n (%)	9 (52.9)	19 (79.2)	0.075
FEV1/FVC < 80%, n (%)	11 (61.1)	17 (58.6)	0.866

Definition of abbreviations: AHI, apnea–hypopnea index; BMI, body mass index; COPD, chronic obstructive pulmonary disease; DLCO, diffusing capacity of lung for carbon monoxide; FEV1, forced expiratory volume in one second; FVC, forced vital capacity; LTOT, long-term oxygen therapy; SMWD, six-minute walking distance; WHO, World Health Organization. Continuous data are presented as mean and standard deviations, and categorical data are presented as numbers (percentage).

**Table 2:** Polysomnography and right heart catheterization measurements of the study population.

	TS90% < 20% *n* = 19	TS90% ≥ 20% *n* = 31	*p* Value
Polysomnographic variables			
TST, min	382.2 ± 27.7	383.6 ± 45.0	0.907
Sleep Efficiency, %	88.7 ± 5.6	86.1 ± 9.0	0.235
N3 Sleep Stage, % of TST	28.9 ± 12.8	29.9 ± 9.9	0.752
REM Sleep Stage, % of TST	9.9 ± 7.4	11.0 ± 7.4	0.637
AHI, events/h	14.3 ± 9.1	25.2 ± 17.9	0.017
OAI, events/h	1.9 ± 3.3	4.9 ± 11.8	0.092
CAI, events/h	0.4 ± 0.6	0.7 ± 2.8	0.523
ODI, events/h	12.0 ± 7.6	22.8 ± 17.7	0.015
Average SaO_2_, %	93.2 ± 1.9	85.6 ± 4.0	<0.001
Nadir SaO_2_, %	83.4 ± 7.9	72.0 ± 9.6	<0.001
Pulmonary hemodynamics on RHC			
mPAP, mmHg	36.1 ± 13.6	48.9 ± 17.1	0.010
PVR, wood unit	5.4 ± 4.9	9.7 ± 5.1	0.004
PAWP, mmHg	12.1 ± 5.7	11.5 ± 6.2	0.387
Cardiac Output, L/min	5.1 ± 1.7	4.6 ± 1.3	0.204
Cardiac Index, L/min/m²	2.8 ± 0.6	2.4 ± 0.7	0.093
SpO_2_, %	95.5 ± 3.0	90.6 ± 4.5	0.001

Definition of abbreviations: AHI, apnea–hypopnea index; CAI, central apnea index; mPAP, mean pulmonary artery pressure; OAI, obstructive apnea index; ODI, oxygen desaturation index; PAWP, pulmonary artery wedge pressure; PVR, pulmonary vascular resistance; RHC, right heart catheterization; SaO_2_, oxyhemoglobin saturation; TS90%, time spent below 90% oxyhemoglobin saturation; TST, total sleep time. Continuous data are presented as mean and standard deviations, and categorical data are presented as numbers (percentage).

**Table 3:** Demographic and clinical characteristics of the patients with OSA vs. ISRH.

	OSA *n* = 27	ISRH *n* = 11	*p* Value
Age, years	57.9 ± 13.1	50.5 ± 17.2	0.156
Male sex, n (%)	13 (68.4)	23 (69.7)	0.960
BMI, kg/m^2^	29.6 ± 5.1	26.2 ± 3.7	0.053
Obesity, n (%)	12 (44.4)	1 (9.1)	0.060
Current smoker, n (%)	2 (7.4)	1 (9.1)	1.000
Asthma or COPD, n (%)	8 (29.6)	3 (27.3)	1.000
Diabetes mellitus, n (%)	5 (18.5)	2 (18.2)	1.000
Hypertension, n (%)	14 (51.9)	2 (18.2)	0.078
Coronary heart disease, n (%)	4 (14.8)	3 (27.3)	0.390
Valvular heart disease, n (%)	2 (7.4)	2 (18.2)	0.564
Atrial fibrillation, n (%)	2 (7.4)	2 (18.2)	0.564
Pulmonary vasodilator use, n (%)	10 (37.0)	4 (36.4)	1.000
Awake SpO_2_, %	93.9 ± 2.0	88.9 ± 4.7	<0.001
LTOT, n (%)	2 (7.4)	2 (18.2)	0.291
WHO function class	1.89 ± 0.64	1.91 ± 0.83	0.936
ESS score	5.4 ± 3.6	4.7 ± 4.4	0.621
mPAP, mmHg	43.3 ± 18.9	51.5 ± 13.6	0.021
PVR, wood unit	7.6 ± 5.5	11.0 ± 4.2	0.049
PAWP, mmHg	12.1 ± 6.7	12.1 ± 4.5	0.993
Cardiac Output, L/min	4.9 ± 1.5	4.3 ± 1.4	0.220
Cardiac Index, L/min/m²	2.6 ± 0.8	2.3 ± 0.5	0.155
SpO_2_, during RHC %	91.5 ± 5.0	92.6 ± 3.4	0.526

Definition of abbreviations: ESS, Epworth Sleepiness Scale; ISRH, isolated sleep-related hypoxemia; mPAP, mean pulmonary artery pressure; OSA, obstructive sleep apnea; PAWP, pulmonary artery wedge pressure; PVR, pulmonary vascular resistance; RHC, right heart catheterization; SpO_2_, oxyhemoglobin saturation; WHO, World Health Organization.

**Table 4:** Unadjusted ORs (95% CIs) for variables associated with severe nocturnal hypoxemia.

		Bounds for 95% CI		
	OR	Lower	Upper	*p* Value
Age	1.063	1.016	1.111	0.007
Male sex	0.969	0.284	3.302	0.960
BMI	0.998	0.876	1.113	0.837
Obesity	1.333	0.375	4.742	0.657
Current smoker	1.241	0.105	14.697	0.864
Asthma or COPD	9.900	1.160	84.471	0.036
Diabetes mellitus	2.479	0.458	13.434	0.292
Hypertension	2.306	0.666	7.986	0.187
Cardiac disease	0.844	0.269	2.647	0.771
Pulmonary vasodilator use	0.868	0.272	2.778	0.812
OSA	6.229	1.770	21.920	0.004
AHI	1.075	1.006	1.150	0.033
ODI	1.084	1.005	1.168	0.036
TST	1.001	0.986	1.016	0.904
N3 sleep stage	1.009	0.957	1.064	0.746
REM sleep stage	1.020	0.942	1.104	0.630
Awake SaO_2_	0.687	0.549	0.860	0.001
Impaired FEV1/FVC	0.902	0.271	2.998	0.866
Impaired DLCO	3.378	0.858	13.296	0.082
mPAP	1.057	1.011	1.106	0.016
PAWP	0.985	0.892	1.088	0.768
PVR	1.216	1.050	1.407	0.009
Cardiac Output	0.768	0.511	1.155	0.204
Cardiac Index	0.473	0.194	1.149	0.098

Definition of abbreviations: AHI, apnea–hypopnea index; BMI, body mass index; CI, confidence interval; DLCO, diffusing capacity of lung for carbon monoxide; FEV1, forced expiratory volume in one second; FVC, forced vital capacity; mPAP, mean pulmonary artery pressure; ODI, oxygen desaturation index; OR, odds ratio; OSA, obstructive sleep apnea; PAWP, pulmonary artery wedge pressure; PVR, pulmonary vascular resistance; REM, rapid eye movement; SaO_2_, oxyhemoglobin saturation; sPAP, systolic pulmonary arterial pressure; TST, total sleep time.

**Table 5:** Adjusted ORs (95% CIs) for variables associated with severe nocturnal hypoxemia.

Variables		Bounds for 95% CI		
	OR	Lower	Upper	*p* Value
mPAP	1.113	1.023	1.210	0.012
Age	1.076	1.007	1.150	0.031
Male sex	0.195	0.025	1.502	0.117
BMI	0.773	0.604	0.989	0.040
Asthma or COPD	13.961	0.958	203.465	0.054
AHI	1.172	1.015	1.353	0.012

Definition of abbreviations: AHI, apnea–hypopnea index; BMI, body mass index; CI, confidence interval; mPAP, mean pulmonary artery pressure; ODI, oxygen desaturation index; OR, odds ratio; OSA, obstructive sleep apnea; PAWP, pulmonary artery wedge pressure; PVR, pulmonary vascular resistance; REM, rapid eye movement; SaO_2_, oxyhemoglobin saturation; sPAP, systolic pulmonary arterial pressure; TST, total sleep time.

## Data Availability

Data collected for the study, including deidentified individual participant data, will be made available to others within 6 months after the publication of this article, as will additional related documents (study protocol, statistical analysis plan, and informed consent form) for academic purposes (e.g., meta-analyses) upon request to the corresponding author (yuksel.peker@lungall.gu.se) and with a signed data access agreement.
